# Hydrocarbon Stapled Antimicrobial Peptides

**DOI:** 10.1007/s10930-018-9755-0

**Published:** 2018-01-12

**Authors:** Dorian Migoń, Damian Neubauer, Wojciech Kamysz

**Affiliations:** 1 Department of Inorganic Chemistry, Faculty of Pharmacy, Medical University of Gdańsk, Al. Gen. J. Hallera 107, 80-416 Gdańsk, Poland; 2 Polpharma Biologics, Gdańsk, Poland

**Keywords:** Antimicrobial peptides, Stapled peptides, Antimicrobial agents, Hydrocarbon stapled, Peptide drugs, Antibiotics

## Abstract

Antimicrobial peptides are promising candidates for anti-infective pharmaceuticals. Unfortunately, because of their low proteolytic and chemical stability, their usage is generally narrowed down to topical formulations. Until now, numerous approaches to increase peptide stability have been proposed. One of them, peptide hydrocarbon stapling, a modification based on stabilizing peptide secondary structure with a side-chain covalent hydrocarbon bridge, have been successfully applied to many peptides. Moreover, constraining secondary structure of peptides have also been proven to increase their biological activity. This review article describes studies on hydrocarbon stapled antimicrobial peptides with respect to improved drug-like properties.

## Introduction

Increasing resistance to antibiotics among microorganisms is a serious threat for human health. According to the Review on Antimicrobial Resistance, by the year 2050 nearly ten million annual deaths will be caused by antibiotic-resistant bacterial infections [1]. Nevertheless, only seven new antibiotics were approved by FDA within the past 10 years. In comparison, some 30 years ago about four new antibiotics were introduced each year. Current unsatisfactory situation is a result of complex processes. Undoubtedly, it is associated with a worldwide overuse of antibiotics in humans and animals resulting in selection of drug-resistant strains [2]. Another crucial aspect is a shift of interest of pharmaceutical industry from antibiotic development to much more profitable chronic disease medications [3]. In view of those facts it is essential to develop effective antimicrobials with novel modes of action to overcome microbial resistance.

Antimicrobial peptides (AMPs) are compounds widely distributed in nature, e.g. bacteriocins, or as components of innate immunity. Generally, they are small up to medium-size (< 10 kDa), amphipathic molecules with a substantial portion (≥ 30%) of hydrophobic residues and an overall positive charge [4, 5]. Hence, AMPs interact electrostatically with the negatively charged microbial surface, penetrate into its membrane lipids usually adopting a specific secondary structure and permeabilize it. This ultimately leads to membrane disruption and bacterial cell death. Depending on the mechanism of action three main models of AMPs antimicrobial activity, barrel-stave, toroidal pore, and carpet-like, have been proposed. Under the barrel-stave model peptides form a pore in the bacterial membrane to which they are oriented perpendicularly. Hydrophobic face of helical peptide is directed towards the membrane and hydrophilic face towards the center of newly formed pore. According to toroidal model pore is formed as a result of peptide-induced lipid bilayer bending. In this case pore line is formed not only by peptides, but also lipid head groups. Lastly, carpet-like or detergent-like model is based on peptide accumulation parallel to membrane surface. When critical concentration of surface accumulated peptide is exceeded, membrane structure is altered, leading ultimately to membrane disruption. Furthermore, AMPs can exert antimicrobial effects via other mechanisms such as inhibition of cell wall synthesis, inhibition of nucleic acid and protein synthesis, or induction of apoptosis/necrosis [6]. Interestingly, for several AMPs an antimicrobial mechanism based purely on microbial process inhibition, with no membrane disruption have been observed. Hypothetically, those AMPs with cell penetrating peptide (CPP) properties may provide a new delivery systems for known drugs [7, 8].

AMPs display activity against broad-spectrum of pathogens such as Gram-positive and Gram-negative bacteria, fungi, parasites, and viruses [9]. As a result, they provide an interesting alternative for conventional antibiotics [10]. At the moment, several antimicrobial peptides are under clinical trials. One of those is pexiganan—a 22-amino acid residue analog of magainin in the form of 0.8% cream, which is in phase III clinical trials for patients with diabetic foot ulcers. Another example is an aqueous topical gel containing omiganan—a 12-amino acid residue analog of indolicidin. Omiganan is also in phase III clinical trials for rosacea [11].

Nevertheless, peptides have some limitations for practical use. First of all, AMPs are prone to enzymatic degradation. This drawback is particularly frequent in case of peptides characterized by flexible conformation in which vulnerable groups are exposed to protease action. Moreover, unsuitable temperature or pH may also negatively affect peptide stability. As a result, AMPs suffer from poor bioavailability and their clinical use is essentially limited to topical formulations. Additionally, environmental conditions (for example: high/low salt concentration or pH) may have a significant impact on peptide secondary structure, and consequently antimicrobial activity [12, 13]. Therefore, modification of AMPs to enhance their stability and antimicrobial activity is mandatory [14].

**Fig. 1:**
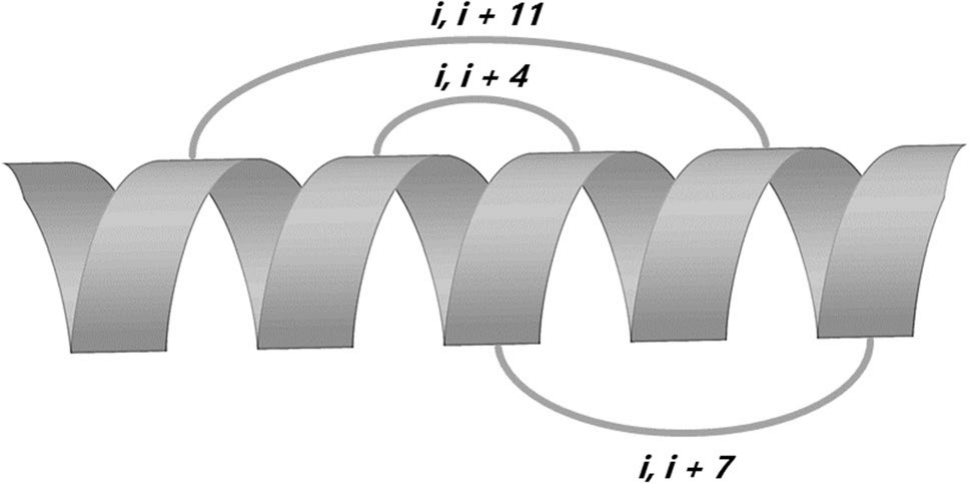
Exemplary locations of staples in helical peptide

Nowadays, many approaches have been proposed to improve serum stability and to increase antimicrobial activity [15–17], inter alia, d-amino acid substitution [18] or other non-ribosomal amino acid substitution [19], PEGylation [20], lipidization [21], and dimerization [22]. Another AMP design approach is peptide stapling. This modification is generally based on formation of a covalent bridge between side-chains. In effect, the peptide is partially protected from enzymatic degradation and its active conformation is stabilized. Therefore, stapled peptide has potentially greater drug-like properties than the parent molecule. According to the bridge chemistry, we can distinguish, among others, hydrocarbon, triazole, azobenzene, thiol-based, lactam, hydrazine, and thioether stapling. Each stapling type may cause different effect on peptide stability and bioactivity [23]. Staples have to connect two side chains located on the same face of the helix to stabilize peptide secondary structure (Fig. 1). In practice these two residues need to be located at i and i + 3, i + 4 (one-loop staple), i + 7 (two-loop staple), or i + 11 (three-loop staple) positions since α-helix has 3.6 residues per turn. Moreover, introduction of two staples (‘stitched peptides’) may enhance protease resistance, improve pharmacokinetic properties and biological activities [24].

Stapled peptides have found application in numerous scientific areas. Many intracellular molecular targets, such as interacting proteins or transcription factors, formerly thought to be “undruggable”, have been effectively inhibited by stapled peptides. This state of affairs was due to the fact that binding interfaces of helical protein fragments are usually too large to be effectively disrupted by small molecules. On the other hand, the binding site is unreachable for biological drugs. Stapling techniques opened a window of opportunity for peptides to overcome those limitations. Moreover, stapled peptides were also applied as receptor agonists/antagonists, enzyme or efflux pump inhibitors, and as agents enhancing cholesterol efflux [25]. Undoubtedly, desirable activity and stability are crucial features of antimicrobial peptides. Therefore, several scientific teams undertook the subject of constraining AMPs α-helical structure.

This review focuses on the latest achievements in the development of hydrocarbon stapled antimicrobial peptides. The article presents some aspects of design and synthesis of the peptides as well as their effects on overall biological activity, stability, and structure.

## Hydrocarbon Stapled Antimicrobial Peptides

### Hydrocarbon Stapling Technique

Hydrocarbon stapled peptides were first introduced by Schafmeister et al. in 2000. Several unnatural C^α^-methylated amino acids with either R or S absolute configuration at C^α^ position and olefinic side chain of different lengths were designed to be integrated into RNAse A in order to single out the most suitable ones for stapling. Their study has shown that R_i, i+7_S with 11-carbon cross-link was the most efficient approach to stabilize α-helical structure and enhance stability towards trypsin enzymatic degradation. Moreover, α-methylation of particular amino acids provided additional, beside the hydrocarbon bridge, so-called helix-stabilization effect [26]. Since then, hydrocarbon stapling has become the most popular stapling technique with several compounds of therapeutic potential in areas of oncology, diabetes, cardiology, and HIV therapy [27, 28].

**Fig. 2:**
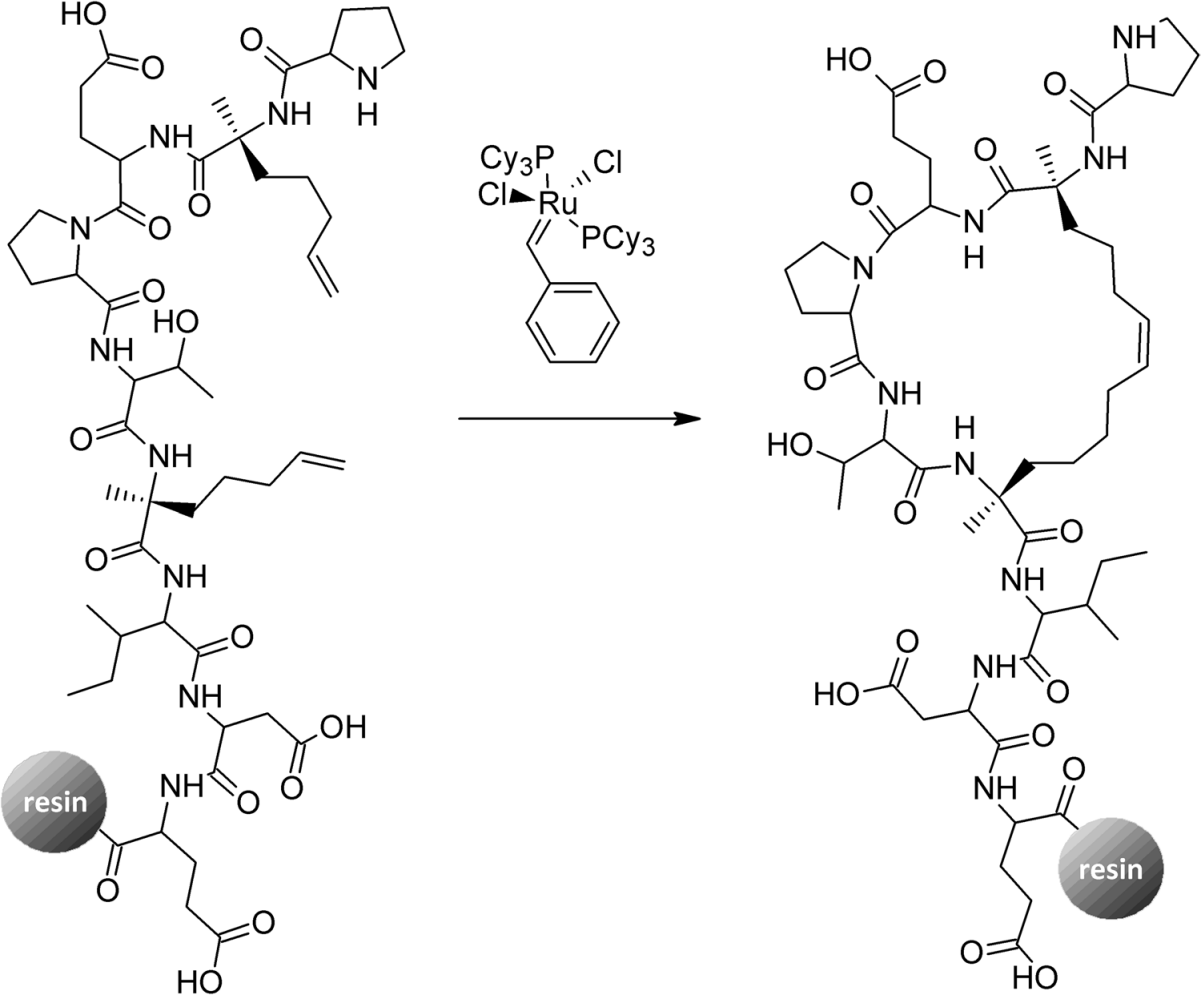
Schematic presentation of hydrocarbon stapling of PXEPTXIDE peptide using a ruthenium-catalyzed ring closing metathesis (X stands for C^α^-methyl, C^α^-alkenyl amino acid residue). Grubbs catalyst—benzylidenebis(tricyclohexyl-phosphine)-dichlororuthenium

Generally, hydrocarbon stapling is performed through incorporation of two C^α^-methyl, C^α^-alkenyl amino acid residues during standard solid-phase peptide synthesis with subsequent ruthenium-catalyzed metathesis macrocyclization reaction of the resin-bound peptide (Fig. 2). Up to date, several ring-closing metathesis catalysts have been developed and applied to peptide hydrocarbon stapling. However, the most commonly used catalyst, are still those introduced by Grubbs and coworkers (for example Grubbs’ first generation catalyst presented in Fig. 2). Additionally, due to the fact that conventional metathesis catalyst are water-insoluble, a growing interest in water-soluble derivatives is observed [29–32]. As helix stabilization of a given stapling depends notably on absolute configuration at C^α^ position and staple length, these features have been exhaustively studied. A brief summary in Table 1 presents optimal parameters for a helix stabilization.

**Table 1:** Optimal parameters for helix stabilization

Stapling type	Chirality of AA at *i*	Chirality of AA at *i* + n	Staple length
*i*, *i* + 3	R	S	6/8
*i*, *i* + 4	S	S	8
*i*, *i* + 7	R	S	11

**Fig. 3:**
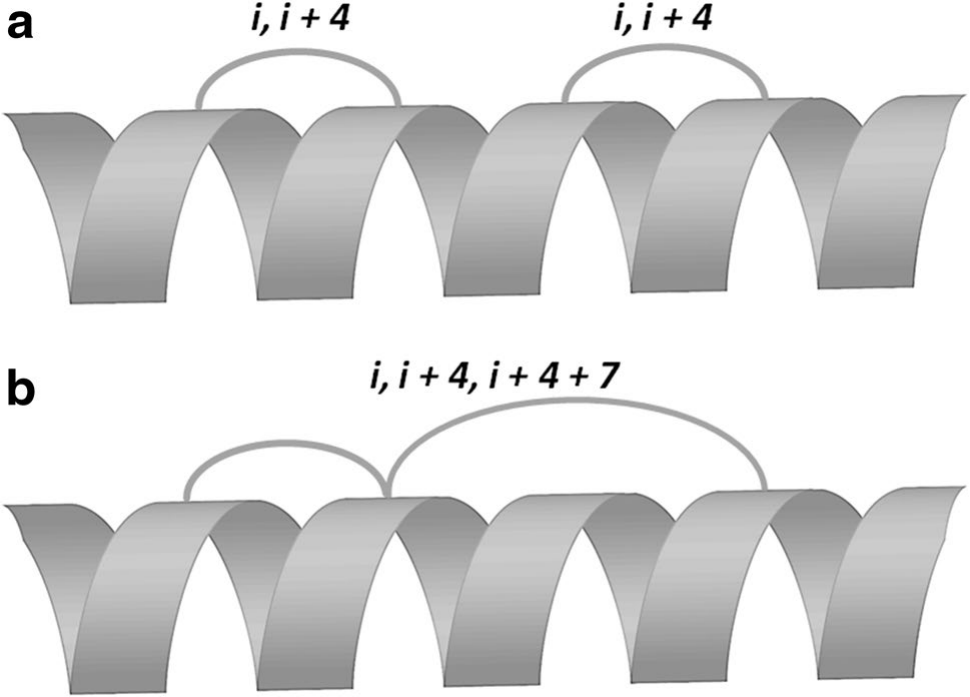
Exemplary locations of staples in double-stapled peptide **a** peptide stapled with two independent staples, **b** peptide stapled in a *i*, *i* + 4, *i* + 4 + 7 manner

Furthermore, peptide may be stapled with more than one stapling. Two main approaches are applied to obtain double-stapled peptides. Longer peptides are stapled either with two independent staples (Fig. 3a) or with the use of 2,2-bis(4-pentenyl)glycine to form a spiro-bicyclic ring connection in i, i + 4, i + 4 + 7 manner (Fig. 3b) [23–25, 33, 34].

In fact, there is a variety of stapled peptide building blocks. These unusual amino acids can be divided into three groups: glycine derivatives, alanine derivatives, and derivatives with two alkenyl moieties. Examples of building blocks are presented in Table 2.

**Table 2:** Alkenyl building blocks for stapled peptides

Description	Structure
Glycine derivatives	
From allylglycine to 2*-*(7′-*octenyl*)*glycine*	
Alanine derivatives	
From 2-(2′-propenyl)alanine to 2-(7′-octenyl)alanine (R and S chirality)	
Derivatives with two alkenyl groups	
From 2,2-bis(2-propenyl)glycine to 2,2-bis(4-pentenyl)glycine	
2-Amino-2-(pent-4-enyl)dec-9-enoic acid	

### Hydrocarbon Stapled Antimicrobial Peptides—Academic Development

Hydrocarbon stapling approach has been described in several scientific articles as a mean to improve AMPs druggability.

Chapuis et al. designed single- and double-stapled analogs of two α-helical peptides of wild bee venom, lasiglossin III (LL-III) and melectin (MEP). To maintain lysine-based hydrophilic domain, several different staples were incorporated along the hydrophobic face of α-helix for both peptides (Table 3). Three different hydrocarbon stapling approaches were chosen: single S_i, i+4_S with 8-carbon cross-link, single R_i, i+7_S with 11-carbon cross-link or two S_i, i+4_S with 8-carbon cross-links. Staple incorporation generally increased helicity in water and proteolytic stability of peptides. Simultaneously this modification resulted in a suppressed antimicrobial activity against pathogenic bacteria and *Candida albicans*. The Authors attributed this to the reduced peptide flexibility. Moreover, stapled analogs were more hemolytic than original molecules. As a result, hydrocarbon stapling of LL-III and MEP did not improve their pharmacological qualities [35].

**Table 3:** List of hydrocarbon stapled antimicrobial peptides studied in articles cited in this review (part 1)

Article title	Peptide	Peptide sequences	Ref.
Effect of hydrocarbon stapling on the properties of α-helical antimicrobial peptides isolated from the venom of hymenoptera		Lasioglossin III analogs	[35]
	LL-IIIs-1	VNWKK**X**LGK**X**IKVVK-NH_2_	
	LL-IIIs-2	VNWKKILGK**X**IKV**X**K-NH_2_	
	LL-IIIs-3	V**X**WKK**X**LGKIIKVVK-NH_2_	
	LL-IIIs-4	VN**X**_**1**_KKI**X**_**1**_GK**X**_**2**_IKV**X**_**2**_K-NH_2_	
	LL-IIIs-5 cis	VN**X**KKILGK**X**IKVVK-NH_2_ cis	
	LL-IIIs-5 trans	VN**X**KKILGK**X**IKVVK-NH_2_ trans	
	LL-IIIs-6 a	VN**X**_**1**_KKI**X**_**1**_PK**X**_**2**_IKV**X**_**2**_K-NH_2_ a	
	LL-IIIs-6 b	VN**X**_**1**_KKI**X**_**1**_PK**X**_**2**_IKV**X**_**2**_K-NH_2_ b	
		Melectin analogs	
	MEP-Ns-1	GFLSILKKVLPK**X**NleAH**X**K-NH_2_	
	MEP-Ns-2	GFLS**X**LKK**X**LPKVNleAHNleK-NH_2_	
	MEP-Ns-3	GFLS**X**_**1**_LKK**X**_**1**_LPK**X**_**2**_NleAH**X**_**2**_K-NH_2_	
	MEP-Ns-4 cis	GF**X**SILKKV**X**PKVNleAHNleK-NH_2_ cis	
	MEP-Ns-4 trans	GF**X**SILKKV**X**PKVNleAHNleK-NH_2_ trans	
	MEP-Ns-5	GFLS**X**_**1**_LKK**X**_**1**_LGK**X**_**2**_NleAH**X**_**2**_K-NH_2_	
	MEP-Ns-5x	GFLS**X**_**0**_LKK**X**_**1**_LGK**X**_**1**_NleAH**X**_**0**_K-NH_2_	
	MEP-Ns-6	GFLS**X**_**1**_LKK**X**_**1**_LAK**X**_**2**_NleAH**X**_**2**_K-NH_2_	
	MEP-Ns-6x	GFLS**X**_**0**_LKK**X**_**1**_LAK**X**_**1**_NleAH**X**_**0**_K-NH_2_	
Truncated and constrained helical analogs of antimicrobial esculentin-2EM	E2EM15W-S1	Ac-TLKQF**X**KGV**X**KWLVK-NH_2_	[36]
	E2EM15W-S2	Ac-TLKQF**X**KGW**X**KDLVK-NH_2_	
	E2EM15W-S3	Ac-TLKQW**X**KGV**X**KDLVK-NH_2_	
De novo design and their antimicrobial activity of stapled amphipathic helices of heptapeptides	S1	Ac-K**X**WKA**X**K-NH_2_	[37]
	S2	Ac-K**X**AKW**X**K-NH_2_	
	S3	Ac-K**X**WKL**X**K-NH_2_	
	S4	Ac-K**X**LKW**X**K-NH_2_	
	S5	Ac-K**X**WAK**X**A-NH_2_	
	S6	Ac-K**X**AWK**X**A-NH_2_	
N-Capping effects of stapled heptapeptides on antimicrobial and hemolytic activities	H-S1	K**X**WKA**X**K-NH_2_	[38]
	H-S2	K**X**AKW**X**K-NH_2_	
	H-S3	K**X**WKL**X**K-NH_2_	
	H-S4	K**X**LKW**X**K-NH_2_	

X stands for C^α^-methyl, C^α^-alkenyl amino acid residues (1, 2—stapled; 0—unstapled); a, b—two not identified isomers

Kim and coworkers published a series of 7 articles greatly contributing to the development of the hydrocarbon stapled antimicrobial peptides. In the first one, Pham et al. designed stapled analogs of esculentin-2EM (E2EM), a 37-residue antimicrobial peptide isolated from Korean frog (*Glandirama emeljanovi*). A single i, i + 4 hydrocarbon staple was incorporated into E2EM15W, a 15-amino acid fragment of E2EM with aspartic acid residue substituted with tryptophan (Table 3). Additionally, to examine the influence of tryptophan residue on peptide-membrane interaction, two stapled analogs differing in tryptophan residue position were analyzed. All stapled peptides used in this study showed a significant increase in antimicrobial activity against *Bacillus subtilis* and *Staphylococcus aureus* as compared to the unstapled E2EM15W. Minimal inhibitory concentration for the stapled peptides was in the range of 3.13–6.25 µg/mL for both strains, whereas for unstapled peptide it was 200 µg/mL for *B. subtilis* and 100 µg/mL for *S. aureus*. On the other hand, both the stapled and unstapled peptides did not show any substantial activity against *Staphylococcus epidermidis* and Gram-negative strains. Oct-4-enyl stapling maintained peptide helicity despite shortening of its length and what is more, enhanced proteolytic stability as compared to that of E2EM [36].

Dinh et al. applied hydrocarbon stapling to short AMPs to increase their helicity and antimicrobial activity. Six de novo designed, stapled, N-capped heptapeptides and their unstapled counterparts (Table 3) were subjected to antimicrobial, hemolysis and circular dichroism (CD) assays. CD spectroscopy demonstrated an increased helicity of the stapled peptides. Furthermore, a close correlation between helicity and antimicrobial activity was observed: in most cases stapling enhanced antimicrobial potential. In fact, the designed peptides caused little or no hemolysis. Nevertheless, in case of two stapled peptides, S3 and S4, the most potent antimicrobials in this study, hemolytic activity was slightly enhanced. This phenomenon was explained in terms of a staple-derived stabilization of the helix structure and increased molecule hydrophobicity [37]. The study was followed-up with another article in which Dinh et al. examined the effect of N-acetylation of the previously described stapled heptapeptides on biological activity. The authors hypothesized that an increase in peptide positive net charge, as a result of removal of N-terminal capping, could contribute to their antimicrobial activity. Moreover, N-acetyl capping is also known for its helix stabilization properties, and thus removing it may destabilize peptide secondary structure and as a result reduce antimicrobial activity. The authors prepared 4 analogs with an N-terminal free amino moiety (Table 3) to learn if the hydrocarbon staple would be an effective standalone helix-stabilizing factor. Only in the case of S3, deacetylation proved to be an advantageous modification. The deacetylated analog maintained helicity, exhibited greater antimicrobial activity, and even lowered hemolysis [38].

A study on short stapled AMPs was reported by Luong et al. Peptide S3 was further modified to improve its pharmacological properties. On the basis of previous studies on helix stabilization with two hydrocarbon bridges, the authors designed a series of dimeric S3 analogs with various linkers (Table 4). Usually helix-breaking amino acid residues in the middle of the helical antimicrobial peptide sequence contribute to their low hemolytic activity. Hence glycine and proline were used as linkers. Furthermore, the authors theorized that usage of longer linkers may lead to peptide folding through interaction of hydrophobic faces and thus increase peptide solubility and help to avoid aggregation. To verify this hypothesis, γ-aminobutyric acid (GABA) and β-alanine were applied as linkers. In general, dimerization did not cause any significant increase in helicity. Although peptide dimers exhibited increased antimicrobial and hemolytic activities, they were different for each linker. Peptide 3PR3-X showed the highest selectivity index. The results demonstrate that linker flexibility plays a key role in differentiation between hemolytic and antimicrobial activity in doubly-stapled antimicrobial peptide dimers [39].

**Table 4:** List of hydrocarbon stapled antimicrobial peptides studied in articles cited in this review (part 2)

Article title	Peptide	Peptide sequences	Ref.
Antimicrobial and Hemolytic Activity of Stapled Heptapeptide Dimers	3GL3	Ac-K**X**_**1**_WKL**X**_**1**_K-G-K**X**_**2**_WKL**X**_**2**_K-NH_2_	[39]
	3BA3	Ac-K**X**_**1**_WKL**X**_**1**_K-βAla-K**X**_**2**_WKL**X**_**2**_K-NH_2_	
	3GA3	Ac-K**X**_**1**_WKL**X**_**1**_K-GABA-K**X**_**2**_WKL**X**_**2**_K-NH_2_	
	3PR3-X	Ac-K**X**_**1**_WKL**X**_**1**_K-P-K**X**_**2**_WKL**X**_**2**_K-NH_2_ a	
	3PR3-Y	Ac-K**X**_**1**_WKL**X**_**1**_K-P-K**X**_**2**_WKL**X**_**2**_K-NH_2_ b	
Mono-substitution effects on antimicrobial activity of stapled heptapeptides	ALA (H-S1)	K**X**WKA**X**K-NH_2_	[40]
	LEU (H-S3)	K**X**WKL**X**K-NH_2_	
	VAL	K**X**WKV**X**K-NH_2_	
	ILE	K**X**WKI**X**K-NH_2_	
	NLE	K**X**WK-Nle-**X**K-NH_2_	
	PHE	K**X**WKF**X**K-NH_2_	
	TRP	K**X**WKW**X**K-NH_2_	
	GLU	K**X**WKE**X**K-NH_2_	
	LYS	K**X**WKK**X**K-NH_2_	
Antimicrobial activity and stability of stapled helices of polybia-MP1	MP1S	IDWKK**X**LDA**X**KQIL-NH_2_	[41]
	MP1S-D8N	IDWKK**X**LNA**X**KQIL-NH_2_	
	MP1S-Q12K	IDWKK**X**LDA**X**KKIL-NH_2_	
Antimicrobial activity of doubly-stapled alanine/lysine-based peptides	Ac-SS-14W	Ac-K**X**AKA**X**KKAAKAAWK-NH_2_	[42]
	Ac-DS-14W	Ac-K**X**_**1**_AKA**X**_**1**_KK**X**_**2**_AKA**X**_**2**_WK-NH_2_	
	Ac-DS-12W	Ac-K**X**_**1**_AKA**X**_**1**_KK**X**_**2**_AKW**X**_**2**_AK-NH_2_	
	Ac-DS-5W	Ac-K**X**_**1**_AKW**X**_**1**_KK**X**_**2**_AKA**X**_**2**_AK-NH_2_	
	Ac-DS-2W	Ac-K**X**_**1**_WKA**X**_**1**_KK**X**_**2**_AKA**X**_**2**_AK-NH_2_	
	Su-DS-5W	Su-K**X**_**1**_AKW**X**_**1**_KK**X**_**2**_AKA**X**_**2**_AK-NH_2_	
	H-DS-5W	H-K**X**_**1**_AKW**X**_**1**_KK**X**_**2**_AKA**X**_**2**_AK-NH_2_	
Hydrocarbon-Stapled Lipopeptides Exhibit Selective Antimicrobial Activity	Val-HSLP	Val-WWV**X**ARA**X**RR	[43]
	Cap-HSLP	Cap-WWV**X**AFA**X**RRR	
Influence of hydrocarbon-stapling on membrane interactions of synthetic antimicrobial peptides	S-6K-F17	KKKKKKAAF**X**AWA**X**FAA-NH_2_	[44]
	S-6K-F17-2G	KKKKKKAGF**X**AWA**X**FGA-NH_2_	
	S-6K-F17-3G	KKKKKKAGF**X**AWG**X**FGA-NH_2_	
	S-6K-F17-3GN	KKKKKKNGF**X**AWG**X**FGA-NH_2_	

X – stands for C^α^-methyl, C^α^-alkenyl amino acid residues (1,2 – stapled; 0 – unstapled); a, b – two not identified isomers

Additionally, Luong et al. conducted studies on the structure–activity relationship of stapled antimicrobial heptapeptides. That article reveals the impact of substitution of fifth amino acid residue (Table 4) on molecule conformation, antimicrobial and hemolytic activity. All studied stapled peptides have been shown to adopt α-helical secondary structure. Moreover, stapled peptides with fifth amino acid residue substituted by aliphatic amino acid (leucine, isoleucine, norleucine or valine) were more helical than those substituted by aromatic amino acid residues (phenylalanine or tryptophan). Generally, an increase in side chain hydrophobicity of the fifth amino acid residue resulted in an overall enhancement in antimicrobial and hemolytic activity (in comparison to that of peptide ALA). Interestingly, although peptide LYS exhibited lower antimicrobial activity than peptide ALA against most strains, it demonstrated the strongest antibacterial activity against *Pseudomonas aeruginosa*. Consequently, peptide LYS seems to be a suitable starting point for further development of a selective antibiotic drug. Additionally, the most potent analog, NLE, was subjected to trypsin digestion and it exhibited nearly 16-fold increased stability compared to its unstapled analog [40].

Another implementation of hydrocarbon staple into known antimicrobial peptide as a mean to increase its drug-like properties was described by Luong et al. A single *i, i* + 4 hydrocarbon staple was incorporated into Polybia-MP1, a 14-amino acid reside antimicrobial peptide originally isolated from the social wasp (*Polybia paulista*) venom. Molecules were compared based on antimicrobial and hemolytic activities, secondary structure and proteolytic stability. Additionally, in order to increase peptide net charge, thus possibly increase antimicrobial activity, two stapled analogs with appropriate amino acid substitutions (MP1S-D8N and MP1S-Q12K) were also synthesized (Table 4). Stapling in case of all compounds increased helicity, with MPS1 exhibiting the highest value (96%, 3.7-fold higher than that of MP1). Furthermore, peptide stapling affected peptide resilience against tryptic digestion. MP1S-Q12K was characterized by nearly 69-fold greater stability during proteolytic assay, as compared to MP1 (t_1/2_ was 1446 and 21 min, respectively). Interestingly, analogs were characterized by increased antimicrobial activity only against Gram-positive bacteria: *B. subtilis*, *S. aureus* (MP1S, MP1S-D8N and MP1S-Q12K), and *S.* *epidermidis* (MP1S-D8N and MP1S-Q12K). As predicted, increasing peptide net charge through substituting asparaginate residue with asparagine, or glutamine residue with lysine, led to increase in antimicrobial activity. However, those modifications and/or incorporation of hydrocarbon staple significantly increased hemolytic activity of studied peptides [41].

Dinh et al. examined application of double-stapling in antimicrobial peptides. Peptides containing lysine, alanine, tryptophan, and two *i, i* + 4 staples were designed (Table 4). In this case, staples provide a helical structure and a well-defined hydrophobic face. Additionally, to examine the influence of various factors on peptide biological activity and secondary structure, several variants differing in N-terminal modification and position of tryptophan residue were also synthesized. Antimicrobial activity, hemolysis, helicity, and proteolytic stability of double-, single-, and unstapled peptides were compared in one study. All stapled peptides adopted helical conformation. However, a double-stapled peptide (Ac-DS-14W) had the highest helical content. Furthermore, the Ac-DS-14W exhibited greater antimicrobial activity than its unstapled counterpart. Among studied compounds, single-stapled peptides were more active against Gram-negative bacteria than the double-stapled. Contrarily, in case of Gram-positive bacteria, double-stapled peptides were more active. According to previous studies, hydrocarbon stapled peptides exhibited increased hemolytic activity. However, different positioning of the tryptophan residue and N-terminal capping moiety resulted in H-DS-5W—a double-stapled peptide effective against both bacterial groups and with decreased hemolytic activity. On this basis, a hypothesis has been put forward that structure of hydrocarbon linker is related to peptide selectivity. Generally, single-stapled analogs were more resistant to trypsin digestion than their unstapled counterparts. Moreover, the double-stapled peptide, Ac-DS-14W, exhibited even higher stability to digestion. In fact, more than 85% of Ac-DS-14W remained intact after 60 min of trypsin digestion, whereas single-stapled and unstapled counterparts were completely digested. Overall, in contrast to Chapuis et al., this study demonstrates that double-stapling, as far as appropriate sequences are applied, may improve peptide pharmacological properties [35, 42].

Another intriguing aspect of hydrocarbon stapling is application of this method into lipopeptides. This approach was applied by Jenner et al. into de novo designed lipopeptides (Table 4). Generally, the molecules were highly amphiphilic due to the hydrophobic N-terminus (with fatty acid and several tryptophan residues) and hydrophilic amidated C-terminus (with several arginine residues). A single *i, i* + 4 hydrocarbon staple was implemented. Antimicrobial and hemolytic activity, secondary structure, and membrane permeabilization properties of the stapled lipopeptides and their unstapled counterparts were analyzed. Moreover, relevance of arginine residue inside the macrocycle was evaluated. Although stapling did not stabilize secondary structure, it increased antibacterial activity and membrane permeabilization. The authors assumed that this outcome was due to the restriction of conformations available to the stapled peptide. Another proposed cause is an increased proteolytic stability and, in consequence, longer duration of action. As in the previously described studies, staple incorporation increased hemolytic potential of the analogs. Moreover, Val-HSLP exhibited lower hemolysis than Cap-HSLP. While the authors explained this lower activity with presence of arginine residue inside of the macrocycle, it could also be an outcome of a shorter fatty acid (Val) at N-terminus. Interestingly, stapled analogs exhibited lower antimicrobial activity against *Candida* strains in comparison to that of the original counterpart LP-1. No activity against *Escherichia coli* strain of either the stapled and unstapled peptides was detected [43].

Stone et al. studied influence of single *i, i* + 4 hydrocarbon staple on the 6K-F17 peptide, a 17-residue, de novo designed AMP (Table 4). Similarly to aforementioned lipopeptides, sequence of 6K-F17 was characterized by occurrence of two regions differing in their hydrophobicity. Conversely, in this case N-terminus was positively charged due to several lysine residues and C-terminus was hydrophobic as a result of alanine, phenylalanine, and tryptophan residue clustering. Stapled 6K-F17 (S-6K-F17) and its unstapled counterpart were compared regarding their secondary structure, peptide–membrane interactions, mechanism of action, and antimicrobial and hemolytic activities. Although staple implementation slightly increased antimicrobial activity, a meaningful increase in hemolytic activity was observed. In order to diminish this undesirable feature, three additional stapled analogs with hydrophobic amino acid residues substituted with glycine and/or asparagine were also synthesized. Each subsequent substitution led to decrease in hemolytic activity of stapled peptides, with S-6K-F17-3GN being characterized by highest therapeutic index. In contrast to majority of described hydrocarbon stapled antimicrobial peptides, stapling of 6K-F17 did not result in molecule with significantly increased helicity in aqueous buffer. Additionally, no increase in peptide helicity was observed in SDS micelles. On the other hand, S-6K-F17 exhibited stronger helical character in bilayers composed of *E. coli* lipids than its unstapled counterpart. Authors explained this phenomenon as a result of more rigid and less negatively charged surface of *E. coli* lipid bilayer when compared to SDS micelles. Interestingly, a significant decrease of helical content in case of glycine/asparagine substituted analogs was observed. Adoption of helical structure in the presence of membrane, however, might not be a sine qua non condition for antimicrobial activity, as it remained at approximately the same level for both stapled and unstapled analogs. Furthermore, S-6K-F17 was characterized by greater depth of tryptophan residue penetration into both SDS micelles and *E. coli* lipid bilayers when compared to its unstapled parent molecule. On the other hand, substituting S-6K-F17 hydrophobic residues with glycine and/or asparagine residues resulted in molecules with diminished tryptophan burial. Finally, NMR studies revealed that both unstapled and S-6K-F17-3GN peptides, despite exhibiting significant differences regarding secondary structure and tryptophan burial, selectively bind to bacterial membranes [44].

### Hydrocarbon Stapled Antimicrobial Peptides—A Patent Review

In 2017 the Dana-Farber Cancer Institute, Inc. patented 16 staple-modified antimicrobial peptides and their derivatives (Table 5). The patent contains a list of stapled peptides and their chemical structures. Furthermore, the authors described pharmaceutically acceptable salts, pharmaceutical compositions, treatment of bacterial infection (bacteria and bacterial biofilm-related), and methods of killing or inhibiting bacterial growth. As an example of their invention, the authors presented multiple stapled-peptide development approaches. Firstly, they synthesized several pexiganan and magainin II stapled analogs. In general, hydrocarbon stapling increase peptides helicity, enhance antimicrobial and hemolytic activity. Basing on the fact that hemolytic activity is usually related to peptide hydrophobicity, the authors modified a staple of pexiganan analog through Sharpless dihydroxylation reaction. Antimicrobial activity of the hydroxylated analogs was preserved, however, lytic activity against red blood cells (RBC) was slightly reduced. Although diol derivatization was not completely satisfactory, it endorsed hypothesis that hemolysis was related to the peptide hydrophobicity. To enhance hydrophilicity the authors modified the staple by additional positive point charge through Sharpless aminohydroxylation (oxyamination). Unfortunately, no data about biological activity was presented. In the next example of the invention, the authors described principles for designing stapled AMPs with selectivity for bacterial membrane. Peptide library based on magainin II *i, i* + 4 and *i, i* + 7 staple screening was synthesized. Generally, implementing staple in both manners increased antimicrobial activity; however, the i, i + 7 analogs were more active against bacteria and RBC. Nevertheless, i, i + 4 analogs exhibited different pattern of hemolysis dependent on the insertion site of the staple. Generally, the i, i + 4 analogs in which hydrophobic patch discontinuity was preserved provided better membrane selectivity. Then, a stapled analog with the highest selectivity index, Mag(i + 4)15, was further modified by moving positive and negative charged residues along the peptide backbone. In principle, substitution of amino acid residue with lysine resulted in an analog with a decreased hemolytic activity. Importantly, antimicrobial activity of the analogs was dependent on substituted position. Moreover, activity against Gram-positive bacteria was decreased more frequently than against Gram-negative bacteria. Similarly to lysine scanning, glutamic acid scanning also provided analogs with a lower hemolytic activity. However, antimicrobial activity was also reduced in nearly all cases. Additionally, to evaluate the influence of positive charge intensity on antimicrobial and hemolytic activity, several histidine substituted analogs were also synthesized. Generally, substitution with histidine resulted in compounds with an increased antimicrobial activity and lower lytic activity against RBC in comparison to those of the lysine substituted counterparts. Thereafter, impact of helix promoting (alanine and 2-aminoisobutyric acid) and disrupting residues (proline, 4-hydroxyproline, d-alanine and d-lysine) on stapled peptide Mag(i + 4)15 activity was examined. Both helix promoting residues increased antimicrobial and hemolytic activity. In contrast, substitution by proline, 4-hydroxyproline and d-lysine resulted in a decrease in both of these activities. d-Alanine proved to be the most suitable residue to modify the peptide due to the increased antimicrobial activity and decreased hemolysis. Based on the results, the authors designed double-stapled analogs. One of the compounds, Mag(i + 4)2,15(I2K, A9H), exhibited similar antimicrobial activity to that of the Mag(i + 4)15, however, with nearly eliminated hemolytic activity. Finally, the authors applied stapled peptide approach to pleurocidin. As a result, a stapled peptide, Pleu(i + 4) 1,15(A9K), with an increased antimicrobial activity and relatively low hemolytic activity was obtained [45].

**Table 5:** Original AMPs and exemplary sequences of their patented stapled analogs (bolded, underlined letters indicate staple location in exemplary analog)

Peptide	Sequences
Magainin II	GIGKFLHS**A**KKFGKA**F**VGEIMNS
Pexiganan	GIGKFLKKAK**K**FGKAFV**K**ILKK-NH_2_
Pleurocidin	GWGSFFKKAAH**V**GKHVGK**A**ALTHYL
Pardaxin	GFFALIPKIIS**S**PLFKTL**L**SAVGSALSSSGEQE
HFIAP	GFFKKAWRKVKHA**G**RRVLKK**G**VGRHYVNNWLK
PGQ	GVLSNVIGYLKKL**G**TGALNA**V**LKQ
Buforin II	TRSSRAGLQFP**V**GRVHRL**L**RK
Dermaseptin	ALWKTMLKKLGTM**A**LHAGKA**A**LGAAADTISQGTQ-NH_2_
Caerin 1.8	GLFKVLGSVAKHL**L**PHVVPV**I**AEKL-NH_2_
Melittin	GIGAVLKVLTTG**L**PALISW**I**KRKRQQ-NH_2_
Cecropin A	KWK**L**FKKIEK**V**GQNIRDGIIKAGPAVAVVGQATQIAK-NH_2_
Lycotoxin I	KIKWFKT**M**KSIAKF**I**AKEQMKKHLGGE
Styelin B	GFGP**A**FHSVSN**F**AKKHKTA-NH_2_
Clavanin B	VFQF**L**GRIIHH**V**GNFVHGFSHVF-NH_2_
Cathelicidin A (CP-11)	I**L**KKWPWW**P**WRRK-NH_2_
Dermicidin	SSLLEKGLDG**A**KKAVGG**L**GKLGKDAVEDLESVGKGAVHDVKDVLDSVL

Another patent by Dana-Farber Cancer Institute, Inc., also published in 2017, describes staple application in intracellular-targeting antimicrobial peptides (I-TAMPs). Generally, I-TAMPs are subclass of AMPs which exhibit their antimicrobial activity through membrane translocation with subsequent microbial process inhibition, in opposition to regular microbial cell membrane disruption. As a part of document, 13 further stapled-derived antimicrobial peptides, their formulations, modifications and applications as antimicrobial drugs were described. Additionally, due to I-TAMPs ability to penetrate microbial membranes, a concept of connecting them with conventional antibiotics in order to: (I) overcome treatment resistance, (II) provide new delivery system for previously ineffective antibiotics or (III) improve selectivity of toxic molecules towards bacteria, was described. As an example of their invention authors applied peptide stapling to buforin II, a 21-amino acid residue histone-derived AMPs, first isolated from stomach of Asian toad (*Bufo bufo gargarizans*). In order to identify the most pharmacologically efficient staple position, an i, i + 4 and i, i + 7 staple scanning was conducted for buforin II. Obtained analogs were characterized by their antimicrobial activity against *E. coli*, hemolytic activity and helicity. Overall, peptide stapling increased buforin II helicity, with i, i + 7 analogs being characterized by greater α-helical content than their i, i + 4 counterparts. Similar tendency was observed for hemolysis, where i, i + 7 analogs exhibited higher hemolytic activity when compared to i, i + 4 derivatives. Two of synthesized analogs, i.e. BFStap (i + 4)7 and BFStap (i + 4)11, showed a significant increase in antimicrobial activity (nearly 200-fold in comparison to buforin II), while maintaining their hemolytic activity at level comparable to unstapled counterpart [46].

## Conclusions

Antibiotic-resistant bacterial infections are becoming a serious threat for mankind. Hence, there is an urgent need to develop new, efficient drugs being an alternative to conventional antibiotics [47].

Peptide stapling emerged as a promising modification for future peptide drug candidates. Until now, two stapled therapeutics, ALRN-6924, a dual inhibitor of MDM2/MDMX-p53 interaction, and ALRN-5281, a growth hormone-releasing hormone inhibitor, both developed by Aileron Therapeutics, Inc., have been under clinical trials [48]. Over the past years, many different stapled peptides have been developed to be potentially useful in cancer treatment and other pathological diseases [27]. Hydrocarbon stapled AMPs appear to be a promising class of anti-infective agents, mainly because of their possible selective activity against bacteria and low incidence of developing resistance. Importantly, it may also increase antimicrobial activity and peptide helicity. Unfortunately, stapling frequently leads to enhanced hemolysis. This phenomenon is presumably a result of an increased hydrophobicity derived from incorporated staple. Because of that, hydrocarbon stapling alone might be insufficient to provide drug-like properties. However, further peptide modifications such as addition of point charges into stapled peptide sequence (for example through substitution of hydrophobic amino acid residue by lysine residue) or insertion of helix-disrupting residues may result in a satisfactory antimicrobial activity and low activity against RBC.

Additionally, antimicrobial peptide stapling, due to increased resistance to enzymatic and chemical degradation, sheds the green light to novel routes of administration. Intravenous injection of stapled AMPs with selectivity for bacterial cells, could greatly facilitate coping with systemic infections caused by antibiotic-resistant microorganisms [49–51]. Furthermore, peptide stapling enables delivering a selective antimicrobial peptide drug via patient-friendly oral route. For instance, Bird et al. examined a double-stapled peptide SAH-gp41_(626–662)_. After oral administration this peptide achieved a measurable and dose-dependent plasma concentrations in contrast to its unstapled counterpart which was undetected (mouse model) [24].

This article reviews the current state of knowledge of hydrocarbon stapled antimicrobial peptides. Undoubtedly, these compounds appear as a promising class of antimicrobial agents. The facts and possibilities presented in this paper encourage for further studies.
